# Paired field and water measurements from drainage management practices in row-crop agriculture

**DOI:** 10.1038/s41597-022-01358-7

**Published:** 2022-06-01

**Authors:** L. J. Abendroth, G. Chighladze, J. R. Frankenberger, L. C. Bowling, M. J. Helmers, D. E. Herzmann, X. Jia, J. Kjaersgaard, L. A. Pease, B. D. Reinhart, J. Strock, M. Youssef

**Affiliations:** 1 USDA-ARS, Cropping Systems and Water Quality Research Unit, Columbia, MO USA; 2 Iowa State University, Ames, IA USA; 3 Purdue University, West Lafayette, IN USA; 4 North Dakota State University, Fargo, ND USA; 5 Minnesota Department of Agriculture, St. Paul, MN USA; 6 University of Minnesota, Dept. of Soil, Water and Climate, St. Paul, MN USA; 7 North Carolina State University, Raleigh, NC USA

**Keywords:** Environmental impact, Agriculture, Water resources, Hydrology

## Abstract

This paper describes a multi-site and multi-decadal dataset of artificially drained agricultural fields in seven Midwest states and North Carolina, USA. Thirty-nine research sites provided data on three conservation practices for cropland with subsurface tile drainage: saturated buffers, controlled drainage, and drainage water recycling. These practices utilize vegetation and/or infrastructure to minimize off-site nutrient losses and retain water in the landscape. A total of 219 variables are reported, including 90 field measurement variables and 129 management operations and metadata. Key measurements include subsurface drain flow (206 site-years), nitrate-N load (154 site-years) and other water quality metrics, as well as agronomic, soil, climate, farm management and metadata records. Data are published at the USDA National Agricultural Library Ag Data Commons repository and are also available through an interactive website at Iowa State University. These multi-disciplinary data have large reuse potential by the scientific community as well as for design of drainage systems and implementation in the US and globally.

## Abstract (summary)

**Table:** 

Measurement(s)	subsurface drain flow • Nitrate-N concentration • water table • soil nitrate concentration • precipitation • grain yield
Technology Type(s)	pressure transducers within drainage control structure/outlet • automated flow-proportional sampling • pressure transducers installed inside deep wells of perforated PVC pipes • dry combustion • on-site weather station • mechanical harvest with combine
Factor Type(s)	controlled drianage • drainage water recylcing • saturated buffers
Sample Characteristic - Organism	Zea mays • Glycine max
Sample Characteristic - Environment	agricultural field
Sample Characteristic - Location	United States

## Background & Summary

Subsurface drainage is used to remove excess water from the landscape for agronomic fieldwork and to ensure suitable crop growth conditions. However, subsurface drainage in the US Midwest provides a pathway for nitrogen and phosphorus loss into waterways, a major concern in nutrient impacted areas such as the Gulf of Mexico. A comprehensive management approach is increasingly needed given the expected increase in artificially drainage due to climate change^1^.

Conservation practices for drained agricultural landscapes are being developed to mitigate nutrient losses from cropland in the Midwest and other highly drained regions. Among these conservation practices, some have the potential to store or redirect water in the landscape and are promising for increasing the resiliency of cropping systems to climate change.

- Controlled drainage (CD), also known as drainage water management, holds water in the field when drainage is not needed using a water control structure to raise the height of water level outflow^2,3^.
- Saturated buffers (SB) divert a fraction of the nitrate-laden drain flow through riparian buffers as shallow groundwater. The nitrate becomes available for plant uptake and denitrification, thus reducing nitrate loads to surface water^4,5^.
- Drainage water recycling (DWR) stores subsurface drainage water in on-farm ponds or reservoirs until it can be applied through irrigation. This reduces water and nutrient outflows while providing supplemental irrigation^6–8^.

The USDA National Institute of Food and Agriculture (NIFA) funded the “Transforming Drainage” project in 2015 to advance and evaluate these drainage management practices’ productivity and environmental tradeoffs. The multi-state, multidisciplinary team conducted research at 24 sites and gathered historical data (not previously available to the public) from collections starting in 1996 from 15 drainage research sites. The resulting dataset provides a resource for understanding subsurface drainage and the effects of drainage management practices in row crop agriculture. Here we present the data, which have been published in the USDA National Agriculture Library Ag Data Commons repository at data.nal.usda.gov^9^ and are also available at drainagedata.org^10^.

**Fig. 1:**
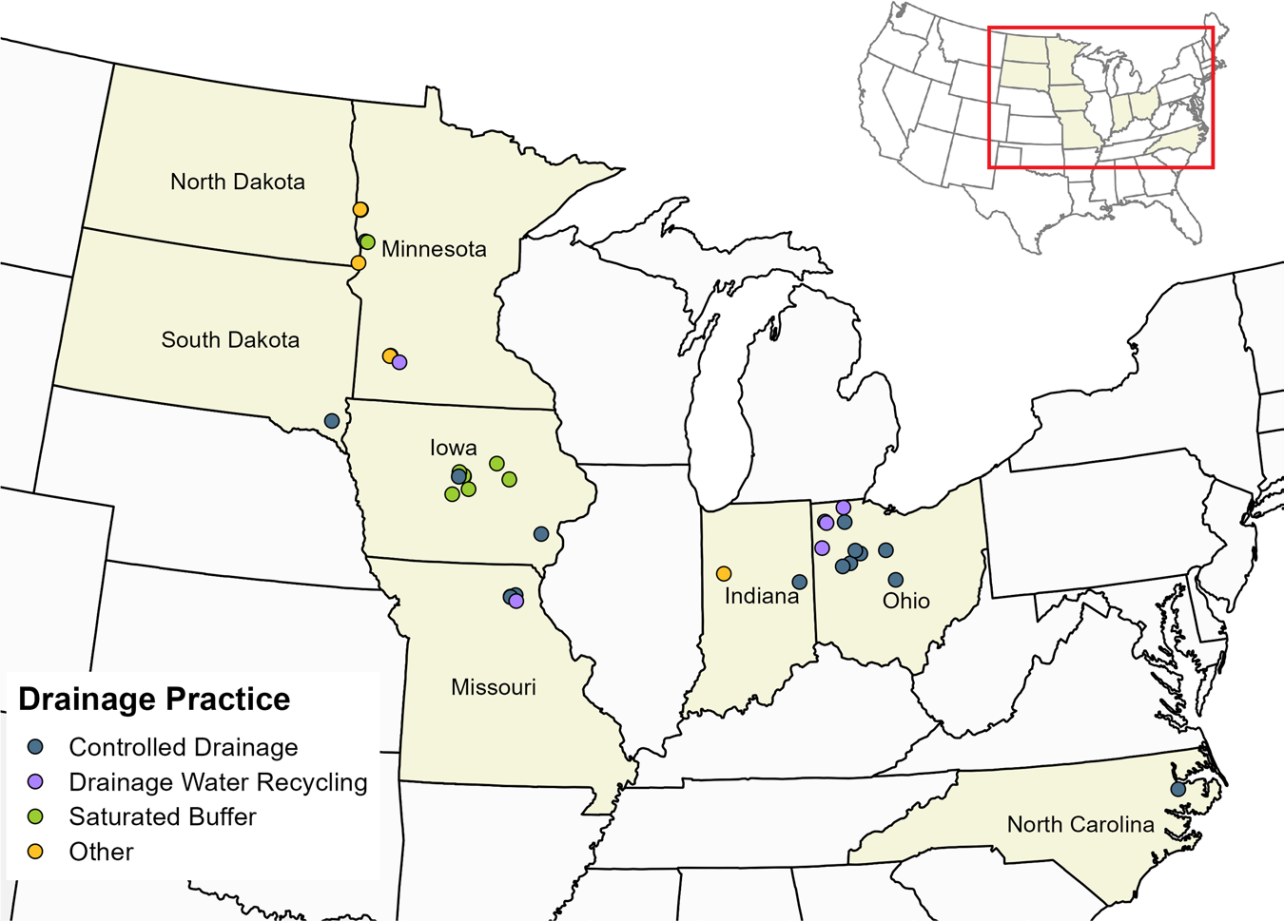
The 39 experimental sites of the Transforming Drainage project. Each drainage practice treatment (controlled drainage, drainage water recycling, saturated buffer, or other) is denoted by colour.

The Transforming Drainage data are unique given the availability of daily, plot-level water quantity and quality measurements paired with agronomic, soil and on-site weather data for artificially drained landscapes. Data allow the study of drainage management practices on water quantity and quality through experimental designs appropriate for each practice. A control-treatment pair design was used for the controlled drainage and drainage water recycling sites (a control plot with uncontrolled drainage and treatment plot employing the drainage management practice) and an upgradient/downgradient design for the saturated buffer sites. Treatments included CD (19 sites), DWR (7 sites), SB (8 sites) and others such as a wetland and undrained reference fields (5 sites) (Fig. 1). The length of data records at each site are 2–17 years, with nine sites including more than 10 years of data.

The 39 experimental sites were located across eight states: Iowa (9), Indiana (2), Ohio (11), Minnesota (9), Missouri (5), North Carolina (1), North Dakota (1), and South Dakota (1). Of the 219 data variables available, key variables (reported in site-years) included tile drainage (206), nitrate concentration (183), water table (92), on-site precipitation (227) and crop yield (201). The data were collected using variable methodologies due to differences in original research goals that dictated the experimental design of each study. Method descriptions are available for each site to improve user understanding of the data.

The data contribute to a more comprehensive understanding of hydrologic, environmental, and agronomic processes in U.S. drained agriculture and globally. Scientists, modellers, and policymakers can gain insights into factors and key drivers (e.g., precipitation patterns, soils, drainage characteristics and cropping systems) behind the agronomic and environmental performance of these drainage practices. Data will be useful for calibrating or validating hydrologic or agroecosystem models. Furthermore, data may be coupled with projections from regional climate models to extrapolate the observed results to climate regimes not included within the study period.

## Methods

### Experimental sites

Experimental designs varied across the 39 research sites with plot size ranging from 0.04 ha to 80 ha. The size of the plot drainage areas varied accordingly from 0.02 to 56 ha. The number of site-years of available data ranging from 2 to 17 with a mean of 7 years. There were diverse soil types, five soil textural classes and soil organic carbon ranging from 0.1% to 3.7%. Corn (*Zea mays*) and soybean (*Glycine max*) were the predominant crops grown, but 23 site-years had popcorn (*Zea mays everta*), wheat (*Triticum aestivum*), forage, oats (*Avena sativa*), or sugar beets (*Beta vulgaris*).

**Fig. 2:**
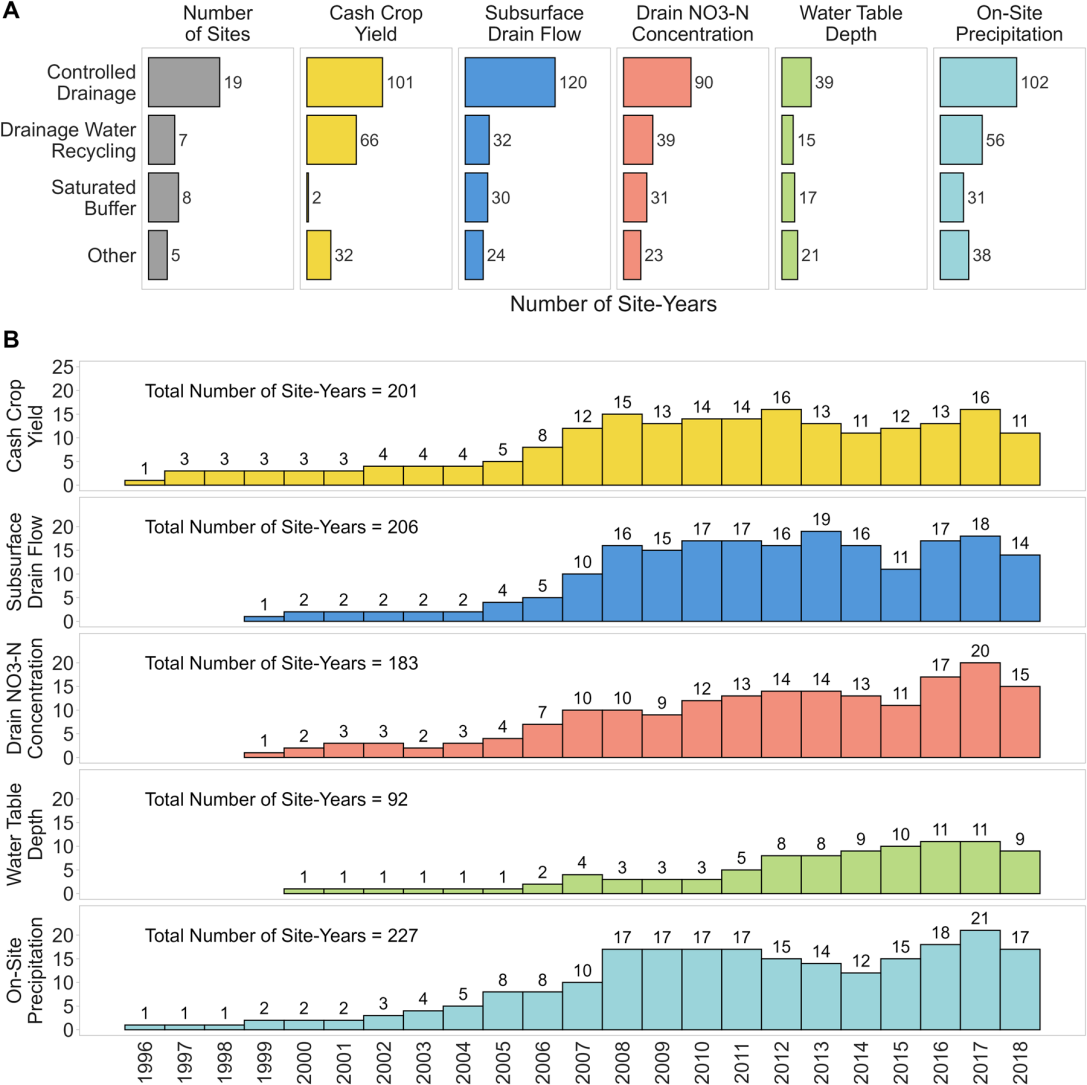
Availability of key variables published in the Transforming Drainage data. Number of site-years shown (**a**) by drainage water conservation practices, and (**b**) by year measurement occurred.

CD was practised at the greatest number (19) of sites (Fig. 2) across seven states in the Midwest and North Carolina. The research sites extended from 35.8° to 46.4° N and 76.7° to 96.9° W. The majority of sites (30) were on private farm (cooperator) fields through a lease or collaborative arrangement, with the remaining 9sites on university-owned and managed research farms. The USDA soil drainage class for the dominant soil type at each site ranged from somewhat poorly drained to very poorly drained^11^. The subsurface drainage of all sites consisted of 102 mm-diameter perforated corrugated tubing except MN_Clay sites (76 mm diameter tubing) and included both CD and free drainage (FD) treatments. Tile depth ranged from 0.61 m to 1.22 m, and tile spacing varied from 6 m to 36 m with median 13.7 m. All sites had similar drain spacings across treatments except IA_di4 and IA_Washington. These two sites varied tile spacing and/or tile depth. IA_di4 tile spacing differed with 27 m and 36 m for FD and CD plots, respectively. While at IA_Washington, tile spacing was 12 m in the shallower drainage treatment compared to 18 m spacing in the conventional drainage treatment. Seven sites had replicated drainage treatments with an average drainage area of 1.1 ha. Sites that did not include replications were larger farm fields with an average drainage area of 10.5 ha, except one university research field with a drainage area of 1.8 ha.

DWR research was conducted at seven sites across the Midwest. Individual research site locations ranged from 39° to 46° N and 83° to 96° W. The treatments at the sites included DWR utilizing controlled drainage with sub-irrigation or controlled drainage with on-surface drip irrigation. In addition, there was a comparison treatment of FD with no irrigation. The three Ohio sites included wetland monitoring in addition to drainage water recycling as part of the Wetland Reservoir Subirrigation System (WRSIS) project^12^.

Eight SB sites were monitored as part of this project, seven in Iowa and one in Minnesota. One of the Iowa sites included the first SB installed in the US^4^. Five sites categorized as ‘Other’ included monitored drainage practices slightly different from the previously described categories. The IN_Tippecanoe site was a wetland with future drainage water recycling planned but not implemented during this period. MN_Clay1 was a conventionally drained farm, MN_Clay3 was an undrained farm with only surface drainage, MN_Redwood2 was an undrained prairie area and ND_Richland had controlled drainage and a sub-irrigated area utilizing a sump pump lift station for water management.

### Data collected at each site

The data describes crop and field management, soil physical characteristics, water quality and quantity time series, drainage system design and specific practice variables for the 39 research sites. Weather data, primarily precipitation and air temperature, were also available for each site. However, other data collected varied since the measurement protocols were not coordinated before research was initiated at many sites. Cumulatively, more than 90 in-field variables were measured across all sites to characterize the performance of these alternative agricultural water management strategies. Water quality and quantity time series (drain flow, water table depth, nitrate-N concentration, and precipitation) were considered essential data for temporal robustness and accuracy regarding the hydrological response.

**Fig. 3:**
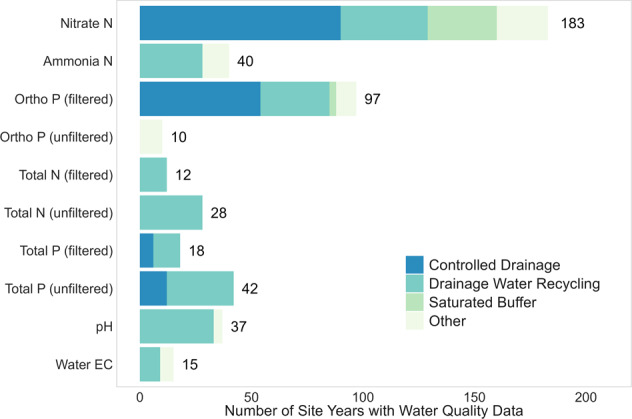
Type of water quality data in the Transforming Drainage data. Number of site-years per variable shown with type of drainage practice denoted by colour. Ortho P, Total N, and Total P are defined by whether the sample was filtered prior to analysis to remove suspended (solid) content from the aqueous fraction.

Precipitation (39 sites) and drain flow or discharge (36 sites) were the most reported variables, followed by nitrate-N concentration (32 sites) and load (30 sites) (Fig. 2). Other common water quality variables are summarized in Fig. 3. In addition, soil moisture time series collected at varying depths were reported for 16 sites.

In addition to the water quantity and quality variables that provide a direct measure of treatment impact to water sustainability, other variables including crop yield, crop and field management and soil characteristic data are important for evaluating inter-site variability. For example, differences in nutrient application with fertilizer and nutrient removal through crop uptake will influence the water quality impact of different treatments. Soil texture (reported for 21 sites), crop yield (29 sites), tillage (27 sites) and fertilizer application (31 sites) were considered most essential of these site characteristic variables^13^. Along with crop yield, sites reported additional variables that assisted in quantifying plant water, nutrient and carbon uptake, including grain moisture content (13 sites), final plant population (end of season plant density; 9 sites), grain total N (8 sites) and grain biomass (6 sites). Whole plant, vegetative and cob biomass, and whole plant, vegetative, cob and grain N and C contents, forage biomass and leaf area index were reported for five or fewer sites.

Sixteen sites reported soil organic carbon and total N, in addition to basic soil texture information. In addition, 31 other soil parameters were reported for a subset of sites; the most common are summarized in Table 1. Soil organic matter, infiltration, lime index, sodium concentration or amount, sodium absorption ratio, neutralizable acid and salinity were reported for five or fewer sites.

**Table 1:** Most reported soil variables and number of sites.

Data Variable	No. Sites Reporting
Soil texture (class, %sand, %silt, %clay)^†^	21
NO_3_ (conc. or amount)	20
Soil organic carbon, total N	16
pH (measured in water or dilute salt solution)	15
K (conc., sat. or amount)	14
Bulk density, Ca, and Mg (conc., sat. or amount)	13
Cation exchange capacity	11
Mehlich or Bray P (conc. or amount)	9
Soil water characteristics	8
NH_4_ (conc. or amount)	7
Hydraulic conductivity	6

^†^All sites have primary, secondary, and tertiary soil series, soil texture, soil drainage class, and soil taxonomic class as reported in the Natural Resources Conservation Service Web Soil Survey^11^; these data are included in the Site Description file (Table 2). Therefore, for sites without soil samples reported at a plot scale, this external data will help inform users.

### Summary of measurement methods

Most experiments were not coordinated when the data collection project was initiated; hence research data collected, length of experimentation, years of available data, and protocols varied. Methods for each research site are provided in the data to document differences in measurement schedule, sample size, sample collection frequency, and equipment precision. Here, we summarize methods for determining drain flow, nitrate-N concentration and load, water table, soil properties and weather data due to the variability across sites within these key metrics. Crop yield is not summarized here despite its importance as a metric due to more consistent methods typically used across sites. Inter-site sampling methods for water measurements varied more than methodology for measuring other parameters. This variability is due to differing infrastructure at each site that required different measurement methods and the financial resources available for monitoring.

#### Drain flow measurement and reporting

Drain flow or discharge data were reported for 36 sites, including 19 CD, eight SB, six DWR and three with other practices (e.g., wetland). For all CD, three DWR and two wetland sites, drain flow was reported in mm/day (drainage discharge normalized by the drainage area). For all other sites, volumetric drainage discharge was reported in m^3^/day. Two of the sites (MN_Clay3 and MN_Redwood2) were undrained control sites that did not report drain flow or discharge. A third site (MO_Shelby) focused on the agronomic impact of subsurface drainage practices and did not monitor drain flow.

Drain flow was measured hourly or sub-hourly at more than 80% of the sites, followed by aggregation to daily flow measurements. Subsurface drainage flow rates were determined as a function of the water head measured using pressure transducers installed inside drainage control structures or at the drain outlet for approximately two-thirds of the sites. The water head was measured upstream of V-notch or rectangular weirs and empirical equations that depend on the weir dimensions were used to determine drain flow, which was measured and recorded hourly or sub-hourly. For IN_Tippecanoe drain flow was estimated as a function of water head using an empirical rating curve. At three sites, drain flow was measured using inline flow meters and recorded by data loggers. The advantage of this method is that flow could be recorded in either direction, valuable for sites experiencing backflow in the drainage system due to high downstream water levels^14^. At ND_Richland, drainage was collected at a sump where a current sensor was used to measure pumping frequency to calculate drainage flow^15^. For an additional three sites, drainage discharge was measured using a depth-velocity meter installed at the outlet of the drainage pipe or a drainage ditch. The drainage discharge was calculated as the product of the flow velocity and the area of flowing water. Only one site (MN_Redwood3) had manual measurements of drain flow that were collected two to three times per week.

Measured drain flow data exhibited variable frequency and duration gaps due to instrumentation malfunctioning, particularly with the automated monitoring systems that provide near-continuous data. Missing data and their non-uniform distribution created problems in statistical analyses when comparing aggregated drain flow and loads from different locations. A systematic approach was used to infill missing drain flow data utilizing variables available at all sites (precipitation, temperature, drain flow) and replicate plots where available. The method consisted of the following three phases and completed in progression, when applicable.

##### Phase 1, fill in zero flow.

During most winters in the northern states, the soil is frozen to the depth of the tile, and no subsurface drain flow is expected. Such periods were identified based on expert judgment by researchers at each site, relying on soil and air temperature information and local knowledge of the drainage system’s response to these conditions. If no drainage measurements were available due to frozen soil, the corresponding gaps in the data record were infilled with zero.

##### Phase 2, predict using replicate plots.

Regression-based estimation was used to infill missing data at three sites which had replicated plots or adjacent fields with available data. Due to the seasonal nature of subsurface drainage from croplands, individual linear regression models were developed for each season: winter (Jan, Feb, Mar), spring (Apr, May, Jun), summer (Jul, Aug, Sep) and autumn (Oct, Nov, Dec). Regression r^2^ values ranged from 0.66 to 0.94 based on the site and season, although mean across-site values were similar: winter (0.80), spring (0.82), summer (0.80), and autumn (0.83).

##### Phase 3, populate based on precipitation and drain flow from the preceding day.

The remaining missing daily drain flow data at 11 sites were filled as described below, based on the assumption that drain flow occurs on a given day only if (a) precipitation occurred on that day or (b) the drain continued to flow from the day before.

- For days with precipitation, a two-day moving average was calculated to account for the time lag between rainfall and resulting drain flow. A linear regression model was fitted to non-zero drain flow and two-day moving average precipitation for each season, with the model’s intercept fixed to zero. We used these models to predict the missing drain flow data for days with non-zero precipitation. The predicted drain flow values were limited to the drainage system’s capacity by replacing predictions greater than the site’s drainage coefficient (depth of water the drainage system could remove within 24 hours) with the coefficient’s value.
- For days with zero precipitation, missing drain flow was calculated from the previous day’s observed flow using the following first-order recession equation

$${Q}_{i}={Q}_{i-1}{e}^{k}$$

where Q is daily drain flow, k is the average recession coefficient of falling limbs calculated as a linear slope of ln(Q), and i indicates day. The recession coefficient was calculated as a linear slope between the peak and inflection point of log-transformed daily drain flow data. The coefficient was calculated for all falling limbs of drain flow data, and the average seasonal values were calculated as their arithmetic mean.

The regression model between on-site precipitation and peak flow and recession equation were only applied to the original (pre-gap-filled) drain flow data. Predictions were not made when the number of missing drainage days exceeded 152 (5 months) within a calendar year; therefore, approx. 18% of the drain flow data remain missing. Both the original and filled data are included in the published data.

#### Nitrate-N concentration and load measurement and reporting

Nitrate-N (NO_3_) concentrations were reported for 32 sites, including 15 CD, eight SB, six DWR, and three sites with other practices (e.g., wetland). The three sites not reporting drain flow (MN_Clay3 and MN_Redwood2, MO_Shelby) did not report NO_3_ concentrations. Two sites (MO_Knox1 and MO_Knox3) provided NO_3_ load along with discharge in place of reporting the concentration of individual water samples. Two sites (OH_Hardin2 and OH_Henry) did not report NO_3_ concentrations or load due to limited water sample collection at these sites.

Six sites collected flow-proportional samples, in which a sample is collected every time a given volume of water passes through the drainage system. The flow-proportional sampling methods at the sites varied. At NC_Washington, a portion of flow was diverted continuously into a composite sample which was collected fortnightly (or more frequently under high flows). At IA_di4, a proportional sample was collected each time the drainage system was pumped. At MN_Redwood1, flow proportional samples were collected during storm and baseflow conditions. These samples were not composited but rather kept discrete. Seven sites used automated samplers to collect time-proportional samples. Five of these sites composited samples daily, while one site (IN_Randolph) collected samples hourly, then combined samples into approximately weekly composites. One site (IN_Tippecanoe) collected weekly grab samples prior to 2016 but then switched to automated, time-proportional sampling composited weekly in March 2016. Sites that used automated samplers typically switched to manual sampling (every two days to weekly frequency) in winter to protect automated samplers from freezing. Twelve sites collected weekly grab samples, another collected samples 2–3 times per week. One site collected biweekly grab samples, and four sites collected grab samples approximately monthly. Regardless of the collection method, all samples were either frozen or refrigerated (4–5 °C) upon return to the laboratory until analysis.

The sampling strategy primarily affects the frequency and compositing strategy of the water samples. Automated samplers permit more complex sampling strategies, such as flow-proportional or sub-daily sampling. However, the disadvantages of this method are the high initial expense of sampling equipment and the propensity for equipment malfunction at below-freezing air temperatures. The potential for equipment failure prompted sites using automated samplers to switch to a manual sampling in winter while drains remained flowing. Manual sampling frequency varied among sites due to differences in site accessibility or personnel availability. Both automated and manual water samples were often composited following collection, and sample compositing frequency ranged from daily to biweekly. Although sample collection frequency and compositing strategy affect the uncertainty of loading measurements, a collection frequency between 3 to 17 days is generally sufficient to reach ± 10% accuracy for annual nitrate load estimation for tile-drained landscapes in the Midwest^16^.

For nitrate-N analysis, 12 sites reported a cadmium reduction followed by a sulfanilamide reaction (equivalent to EPA 353.2). However, there was a slight methodological variation depending on the equipment, either Lachat QuikChem 8000 Flow-Injection Analyzer or SEAL AQ2 Discrete Analyzer. The resulting nitrate-N concentrations calculated via cadmium reduction were directly comparable regardless of the instrument used. At one site, SD_Clay, ion chromatography (EPA 300.1) was used to measure nitrate-N in 2015 but was subsequently switched to a cadmium reduction method. Samples at the seven IA sites were analysed by second-derivative spectrophotometry^17^.

Daily nitrate loads were calculated by multiplying nitrate concentration by drain flow and were therefore available for 32 sites for which both values were reported. Load calculation methods differed slightly in terms of determining the volume of water associated with each concentration. Typically, linear interpolation was used to determine the daily nitrate concentration at sites which collected “grab” water samples following precipitation events or on a schedule spanning two days or more. One variation used assumed the measured concentration was representative of adjacent days (prior and post), hence no interpolation was done. One site (OH_Delaware) used a midpoint approach to determine the time interval in which measured concentrations were associated with, while another site (IA_di4) assumed measured concentrations represented all water drained before the sample was collected.

#### Water table measurement and reporting

The water table was measured at 16 sites including nine CD sites, three SB sites, three DWR sites and one wetland site. Documenting water table fluctuation is key to experimental and modelling research investigating crop production systems on artificially drained soils. In a tile-drained field, the water table is used as an input parameter in estimates of drain flow, evapotranspiration, and soil hydraulic conductivity^14,18,19^. In controlled drainage, the water table is used to determine CD effectiveness and guide water management in the field for different crop stages. For DWR practice, the water table, particularly the midpoint water table, is used to evaluate sub-irrigation performance, such as uniformity and efficiency^20^. In a saturated buffer field, the water table is the most important factor used to indicate a field’s saturation status^21^.

The field water table was typically measured at the midpoint between the subsurface drains. Some field studies also measured the water table at two locations, one near the drain tile and the other at the midpoint between two drain tiles. The water table was commonly measured and recorded hourly or sub-hourly using pressure transducers installed inside 1.5–2.5 m deep wells of perforated PVC pipes. The water table depth was calculated using the measured water pressure above the transducer, and the *in-situ* water temperature and barometric pressure measured in a nearby field and periodically adjusted with manually measured water tables. If there were any discrepancies, all previous water table depth data were moved up or down correspondingly.

Differences across sites spanned the type of pressure transducers used, depth of measurement (1.5 to 2.4 m), data collection frequency (0.17 hr (10 min) to 6 hr), location of the measurement, and the length of the screened section of the pipe. The selection of the transducer type was due to individual choice and cost, while differences in the water table depth measurements were affected by the soil types and drain depth. The frequency of data collection was based on data logger capacity, water table variations, and the purpose of the measurements. The length of the perforated (“screened”) section of the pipe, in which the transducer was installed, also varied. For a typical tile-drained field, the pipe was screened beginning 0.3 m below the soil surface while for a saturated buffer, the pipe was screened beginning at the soil surface^16^. The data collection frequency for the saturated buffer area was every 6 hr since the water table variations were minimal across time. Within the field experiments, data were collected every hour at 10 sites and every 0.17 hr (10 min) at two sites.

#### Soil physicochemical variable measurement and reporting

Potentially important soil physical and chemical properties that might affect or be affected by soil drainage were collected from 19 experiments across six states. Data included 17 total variables, continuous and categorical. Soil physical variables included bulk density, hydraulic conductivity (saturated), moisture (water) content, temperature, texture, and soil water retention data (used to form a water retention curve). The remaining 11 variables were chemical properties. The five most common chemical variables characterized were nitrate, total nitrogen, soil organic carbon, pH and cation exchange capacity with several sites using similar methods^22^.

There was large variability of soil sampling depth among the studies and within specific variables at a site, and in a few cases (<1%) sampling depth intervals were not specified. Of the soil physical properties, soil texture was consistently measured using the same quantitative method (hydrometer). Soil textural class, a categorical variable, was reported once during the study period. Although not explicitly stated, it was assumed that researchers used intact soil cores and standard methods^23^ for quantifying bulk density. Soil water content was measured using capacitance/frequency domain technology or dielectric impedance-based sensor. At sites where soil temperature was measured, a thermistor in thermal contact with a water content probe measured temperature. Soil water content and temperature data were saved at time intervals ranging from five to 60 minutes.

Of the soil chemical properties, total nitrogen was measured once during a study and consistently using the dry combustion method. The soil nitrate concentration measurement frequency was not specified, and nitrate was extracted using potassium chloride (2 M KCl). The nitrate analysis method, when specified, was either by cadmium reduction or nitrate specific electrode. Soil pH was analyzed using a 1:1 soil:water mixture or salt solution. Where soil organic carbon was measured, the dry combustion method was used after carbonate removal by sulfuric acid.

#### Weather data collection

Weather information was available from on-site weather stations and nearby weather stations operated by the Federal Aviation Administration, National Weather Service, or state agricultural weather networks. Precipitation was recorded using automated tipping bucket gauges for rainfall at 31 sites, while at one site, a manual read gauge was used. Multiple rain gauges were installed for redundancy and measurement intercomparisons at four sites. The air temperature was recorded electronically except at one site, where a manual thermometer was used. Weather information was recorded at daily intervals.

Precipitation measurements were a particular focus due to its important effects on drainage response and its higher spatial variability compared to solar radiation, air temperature, humidity, and wind speed. To help improve the quality and integrity of precipitation data in situations where the nearest weather station was located more than 1000 m from the research site or to provide more localized information, on-site precipitation was collected at 20 sites. These onsite precipitation measurements used tipping bucket gauges with 15.2 or 20.3 cm orifices to optimize measurement accuracy. Rain gauges may underestimate rainfall due to wind effects, wetting of and evaporation from the funnel, mechanical limitations, splashing out of the funnel or other factors^24^ by up to 20% at high rainfall intensities^25^. Reference evapotranspiration was estimated for short or tall reference (or both) using the standardized Penman-Monteith^26,27^ equation for most sites, except for three of the sites where potential evapotranspiration was estimated using the temperature-based Thornthwaite equation^28^.

Weather data were reviewed by the Data Management Team using visualization and statistical methods to ensure uniformity and high quality of the data across all sites. Plausible ranges were defined for each parameter based on known climatological limits for the site with guidance by research personnel. For certain parameters, absolute limits were used to identify impossible values (e.g., such as wind directions not between 0 and 360 degree or relative humidity greater than 100%). In addition, an internal consistency check was performed between daily minimum and maximum air temperature observations. All questionable records were flagged, reconciled or removed except for 6 records with internal inconsistency at IN_Randolph but retained and the decision left to data users. The percent of missing data varied among variables (1.2% relative humidity to 36.7% evapotranspiration) and research sites (0 to 38.5% at OH_Crawford). Only, 4.2% of the records are missing in total and it is left to the discretion of users to reconstruct missing data.

### Data ingestion and processing

The team leveraged a previously existing Google Cloud entry and management platform developed for the USDA–NIFA funded Sustainable Corn CAP team^29–31^. The system was expanded and customized to the needs of the Transforming Drainage team. Team members were led through a workflow (Supplementary Fig. 1) utilizing Google Sheets (web browser-based spreadsheets stored in the cloud), customized Google App Scripts (web browser-based data entry forms) and uploading free-form content to the shared Google Drive. All information was stored in Google’s Cloud with associated versioning and metadata tracking with team member identification. Automation was used to provide daily alerts to Data Management Team personnel regarding content modified within the previous 24 hours for further review and quality control. Additional automation via Python scripting synced data content to a local PostgreSQL database server housed at Iowa State University for further analysis, quality control and viewing within custom interfaces on an internal website.

The team implemented a targeted data entry approach to ensure timely revision and sharing of key variables among project members. For this purpose, all variables were categorized as primary or secondary in importance based on research priorities. Primary variables were crop yield, drain flow, tile nitrate-N concentration, water table depth, on-site precipitation, and field management data. These data were critical to address research areas of interest and expected to be the most time-consuming to retrieve, given the sampling frequency and quality control needed. All other variables were secondary and incorporated into the database following the entry of primary variables.

The general workflow of the team included characterizing treatments and data collected for each site, building the data dictionary, creating site-specific spreadsheets for different subsets of variables, entering priority data, entering methods used to collect measurements, entering secondary data, and adding additional research sites from cooperating partners. Subsequently, supporting photographs and drainage system design drawings and site maps were uploaded. Quality control was performed on all data throughout the process. Data were interrogated multiple times during the life of the project, and data reviews with site personnel occurred annually to address issues or questions. Issues needing attention were listed internally for site personnel to further scrutinize data prior to publishing.

Standards were instituted for each variable to ensure uniformity and enable data synthesis across dispersed datasets. These standards specified the minimum frequency for continuous or semi-continuous variables (e.g., drain flow was required to be reported at least daily) and precision according to assigned units of measurement. There was only one site (MN_Redwood3) that did not meet the requirements in terms of periodicity for soil moisture, as measurements were collected weekly. At several locations, variables were reported at higher or irregular frequencies. At the end of the project, all high-frequency measurements were aggregated into values corresponding to the minimum frequency of the respective variable.

Nutrient concentration measurements were limited to the detection limit corresponding to the analytical methods used at each site when the information was available. Any concentration below the limit was replaced with the corresponding limit, such as < 0.3. Concentrations were not adjusted at several sites where the detection limit was not available. Occasionally, zero concentrations were reported, indicating that the analytical method could not detect the nutrient. This limitation should be considered for inter-site water quality analysis.

## Data Records

Data have been published^9^ to the USDA National Ag Library Ag Data Commons (USDA NAL ADC) repository with an assigned Digital Object Identifier (doi) and made available at drainagedata.org. Both sites provide identical copies of the data. Data at USDA NAL ADC are stored as individual .csv files to meet their recommended standards and ensure machine-readability^32^. Each .csv file contains research data or metadata necessary to represent and understand these drainage systems with purposes specific to each file (Table 2).

**Table 2:** Data available at the USDA National Ag Library Ag Data Commons with file names, content, and purpose stated.

File Name on Website	.csv file name^†^	Content	Purpose
Data dictionary	data_dictionary_5	314 data variables with description, units, and acceptable values	Use to interpret the column headers in other data sheets
Site description	meta_site_characteristics_0	Individual site characteristics of 39 experiments (expt)	Understand site establishment, location, drainage infrastructure, soil types from USDA NRCS Web Soil Survey^11^
Plot description	meta_plot_characteristics	Characteristics of 191 individual plots for each expt	Describe plot drainage infrastructure, drainage area and slope
Plot treatment	meta_treatment_identifier	1718 treatment * year assignments for each plot with treatment codes standardized across all expts	Identify expts and plots of interest based on specific interest area or treatment
Methodology	meta_methods	459 methods used in measuring biophysical data across expts	Describe methods used across sites and time that may alter precision, frequency, or presence of data
Drainage data	drain_flow_and_N_loads_data_0	230640 plot * daily entries for drain flow and N load at original and filled (modelled) values. Nitrate-N is on an area basis.	Quantify the amount of water and nitrate-N exported from plots.
Water quality data	water_quality_data	24824 plot * daily nutrient concentrations	Provide concentration of nutrients exported with water from plots
Water table data	water_table_data	223491 plot * daily water table depths	Characterize hydrology of fields or buffers, discern available water for crops and effect of control structures
Water stage data	water_stage_data	2738 measurements of wetland stage depth for 3 Ohio sites only.	Provide an indicator of outflow for wetland sites
Irrigation data	irrigation_data	752 plot * daily irrigation applications at 3 sites that reported daily irrigation amounts	Quantify irrigation water applied to plots at expts with overhead or subirrigation capabilities
Agronomic data	agronomic_data_0	2910 plot * year entries across 17 metrics	Quantify above-ground plant productivity and nutrient composition
Soil moisture data	soil_moisture_data	383154 plot * daily measurements of soil moisture	Quantify available soil moisture at differing depths
Soil physicochemical properties data	soil_properties_data	22750 plot * depth measurements of soil chemical, physical properties	Quantify soil properties soil texture, carbon, bulk density, and nutrient composition
Weather data	weather_data	84550 days of weather data from on-site and nearby weather stations	Use for analyses and modelling as much of this is unpublished on-site weather data that is more site-specific
Field management - drainage water management	mngt_dwm_data	2180 occurrences in which control structures were moved up or down to manage the water level	Provide frequency and timing of control structure alteration
Field management - irrigation	mngt_irrigation_data	771 occurrences of irrigation applied per plot over time at 8 sites	Quantify irrigation water applied to plots in expts with overhead or subirrigation capabilities.
Field management - planting	mngt_planting_data	2629 planting operations at the plot level, including hybrid/variety and seeding rate.	Define the start of the season for managing the cash crop
Field management - harvesting	mngt_harvesting_data	2007 harvest operations at plot level	Defines the end of the season for managing the cash crop
Field management - fertilizing	mngt_fertilizing_data	4801 fertilizer applications to plots	Quantify the amount of fertilizer or manure applied and allocation per nutrient (N, P, K, S, Zn, Mg, Ca, Fe)
Field management - tillage	mngt_tillage_data	1809 tillage operations to plots	Define the type of tillage and intensity performed per plot before or during cash crop
Field management - residue	mngt_residue_data	Whether or not plot was considered “no-till” across 1607 plot * year entries	Use when tillage operations are not reported to know whether operations are missing or did not occur
Field management - notes	mngt_notes	111 observations relevant to the expt, plot, or year	Explain missing or outlier data and items to consider for analyses

^**†**^Four file names end with a number; this denotes an iteration within the repository system but not of concern to users.

## Technical Validation

Data were scrutinized by individual site investigators and centrally by data experts with domain knowledge. Following upload by site investigators, all data underwent centralized validation programmatically and manually. Multiple steps and processes were used in this validation process, including establishing relevant and appropriate bounds based on local conditions, applying centralized standards and uniformity, and harmonization.

Data validation was first performed at the individual site level by personnel with expert knowledge of local conditions. This approach recognizes that the feasible ranges of the measured crop, soil and hydrologic parameters are limited by chemical, biological and physical processes that differ based on local soil, climate, and agronomic management conditions. For example, local precipitation intensity and depth, soil conductivity and drainage system design control the maximum expected daily drain flow rates. Based on local knowledge and conditions, data experts reset observed drain flow to a no data value if recorded values fell outside expectations based on observed precipitation or soil moisture. For example, a drain flow spike was deleted when no precipitation was observed. In addition, pronounced differences in seasonality in drain flow, soil moisture, soil temperature and related variables were used to screen for appropriate values. For example, given the low likelihood of winter drain flow in the northern and western portions of the domain, site experts set missing drain flow data to zero values, following examination of soil temperature and moisture data.

Given the large variation across sites and years in climate, soil, hydrology, and farm management practices, these measured data differed substantially and appropriately. Data distribution was assessed for all numeric measured variables (84 total) across research sites to ensure meaningful value ranges existed (Table 3). When outliers were detected, personnel reviewed past field and laboratory notes to discard or retain. In isolated cases, samples were resubmitted to laboratories for analysis. Individual site measurements were visualized relative to other experimental sites as a visual confirmation of magnitude and range. Management data, including farm operations, were also standardized with validation performed by data personnel knowledgeable about farmer practices and appropriate fieldwork methods as they pertain to crop varieties, fertilizer applications, tillage operations and seeding rates.

**Table 3:** Summary statistics (number of observations (n), mean, first quartile (Q1), median, and third quartile (Q3)) for up to four often reported measurements within each data table.

Table	Variable (units)	n	Mean	Q1	Median	Q3
Agronomic	crop_yield^†^ (kg/ha)	2399	7516	3560	6336	11364
Agronomic	grain_moisture^†^ (g/kg)	2072	143	114	141	170
Agronomic	final_plant_population^†^ (number/ha)	1139	110550	61209	73020	81837
Agronomic	grain_total_N^†^ (kg N/ha)	710	149	121	140	169
Drainage	nitrate_N_load (kg N/ha)	137130	0.07	0.00	0.00	0.03
Drainage	drain_flow (mm/day)	134626	0.98	0.00	0.00	0.53
Drainage	drain_flow_filled (mm/day)	103279	0.71	0.00	0.00	0.50
Drainage	nitrate_N_load_filled (kg N/ha)	83634	0.07	0.00	0.00	0.04
Soil moisture	soil_moisture (cm3/cm3)	307295	0.321	0.263	0.320	0.386
Soil moisture	soil_temperature (deg C)	251017	11.0	3.3	11.3	18.2
Soil moisture	soil_ec (dS/m)	71974	0.090	0.051	0.083	0.118
Soil physicochemical properties	matric_potential (bar)	17561	−1.3	−0.3	−0.1	0.0
Soil physicochemical properties	water_content (cm3/cm3)	15625	0.4	0.3	0.4	0.5
Soil physicochemical properties	NO3_concentration (mg NO3-N/kg)	2155	4.1	1.2	2.6	5.2
Soil physicochemical properties	bulk_density (g/cm3)	1587	1.3	1.2	1.4	1.4
Stage	stage (m)	2738	0.8	0.7	0.7	0.8
Water table	water_table_depth (m)	177928	1.3	0.9	1.3	1.8
Water quality	nitrate_N_concentration (mg NO3-N/l)	19901	7.7	1.0	5.8	11.1
Water quality	ortho_P_filtered_concentration (ug PO4-P/l)	9999	80	15	30	71
Water quality	ammonia_N_concentration (mg NH3-N/l)	6318	0.1	0.0	0.0	0.1
Water quality	total_P_unfiltered_concentration (ug P/l)	6047	225	36	77	220
Weather	precipitation (mm)	82868	2.4	0.0	0.0	0.5
Weather	air_temp_max (deg C)	59288	16.2	6.3	18.2	26.8
Weather	air_temp_min (deg C)	59288	4.6	−3.0	5.3	13.9
Weather	solar_radiation (MJ/m2)	41589	13.6	7.2	12.8	19.9

Statistics on additional measurements are reported in Supplementary Table 1. Metadata describing site characteristics, field operations and non-numeric data are not included here.

^†^Measurements are reported across crops including corn, soybean, wheat, popcorn, sugarbeet, cereal rye and sorghum-sudangrass.

## Usage Notes

The Transforming Drainage dataset is a unique resource for understanding drainage and evaluating the productivity and environmental tradeoffs of drainage storage practices. Although data coverage is entirely in the US, it has far reaching implications in areas with similar soil and hydrological constraints and opportunities. Its use to address questions beyond the scope of the Transforming Drainage team is encouraged such as:

- Model developers can use data to validate algorithms and test the predictive ability of models to improve understanding of hydrologic and agroecosystem processes in drained landscapes. The simulation of conventional drainage could be assessed using the untreated plot or before treatment measurement collection from each site, and the ability of models to predict the effect of conservation practices could be tested. Models have been published with these data to-date^18,33^.
- Tool developers can utilize data for necessary inputs to improve existing or build new applications. A current example is the Evaluating Drainage Water Recycling Decisions (EWDRD) tool^34^ which requires daily drainage data.
- Drainage engineers or managers can use the data as baseline or reference values of typical drain flow and other drainage variables across the region.
- Researchers can use the data in establishing data standards for variable measurement, variable reporting, collection frequency, and unit reporting.
- Researchers can leverage these data with their own or other databases such as STEWARDS (water quality at watershed scale^35^), MANAGE (50-year compilation from literature at the field or replicate scale^36^), and Sustainable Corn CAP Research Data^31^ (five years overlap at 5 sites (IA_Washington, IN_Randolph; MN_Redwood1, MN_Redwood2, OH_Auglaize2).
- Educators can use the drainagedata.org visualization interfaces for undergraduate education, outreach presentations to farmers, and other purposes.

Differences in methods and variations across the sites may present challenges to users and we encourage delving into site details through published metadata and web resources. Extensive metadata are available in .csv format from the USDA National Ag Library Ag Data Commons repository^9^ and the interactive website (drainagedata.org) at Iowa State University^10^. Technical, 2–4-page site summaries and imagery are also available on the project website (transformingdrainage.org)^37^ and drainagedata.org. Finally, published studies for many of the sites are available in the literature (Table 4).

**Table 4:** Site identifier and associated published studies that provide additional information about drainage study plots and analytical methods.

IA_Boone, IA_Hamilton1, IA_Hamilton2, IA_Hamilton3, IA_Story1, IA_Tama^21^
IA_Story2^42^
IA_Washington^43^
IN_Randolph^44,45^
IN_Tippecanoe^45,46^
MN_Clay1, MN_Clay2, MN_Clay3^18,47^
MN_Clay1, ND_Richland^20,48^
MN_Wilkin1, MN_Wilkin2, MN_Wilkin3^49^
MO_Knox1^50,51^
MO_Knox2^52^
MO_Knox3^53,54^
MO_Knox4^55,56^
MO_Shelby^57,58^
NC_Washington^59,60^
ND_Richland1^15,61^
OH_Auglaize1, OH_Auglaize2, OH_Crawford, OH_Defiance1, OH_Hardin1, OH_Hardin2, OH_Henry^62,63^
OH_Defiance2, OH_Fulton, OH_VanWert^12,64^
OH_Delaware^65,66^
SD_Clay^67^

## Supplementary information

Supplemental Information

## Data Availability

Code written by the Data Management Team in support of this project can be found on github.com under an open and permissive usage license. Gap filling of the drain flow data and visualization of the results were performed within RStudio^38,39^. The source code and corresponding input and output data are publicly available on GitHub^40^. The code is split into three pieces corresponding to individual phases of the gap-filling method. In addition, there are separate scripts for the visualization of the data. At drainagedata.org, users can visualize the data with customized tools, query based on specific sites and measurements of interest, and access site photographs, maps, summaries, and publications. The code used for the backend data services and frontend web interfaces can be found on GitHub^41^.
