# Supplementary material for: Paired field and water measurements from drainage management practices in row-crop agriculture
Source: Sci Data. 2022 Jun 1;9:257. doi: 10.1038/s41597-022-01358-7 (PMC9160221; doi:10.1038/s41597-022-01358-7)
Supplement: Supplementary file 1 — Supplemental Information [file 41597_2022_1358_MOESM1_ESM.pdf]

Supplementary Material for:

Abendroth *et al.*, Paired field and water measurements from drainage management practices in row-crop agriculture. *Sci. Data*. (2022).

Table of Contents

- Supplementary Figure 1. Cloud-entry interfaces and overall workflow of team members and data personnel to aggregate, curate, and publish experimental data.
- Supplementary Table 1. Summary statistics for variables not included in Table 3.

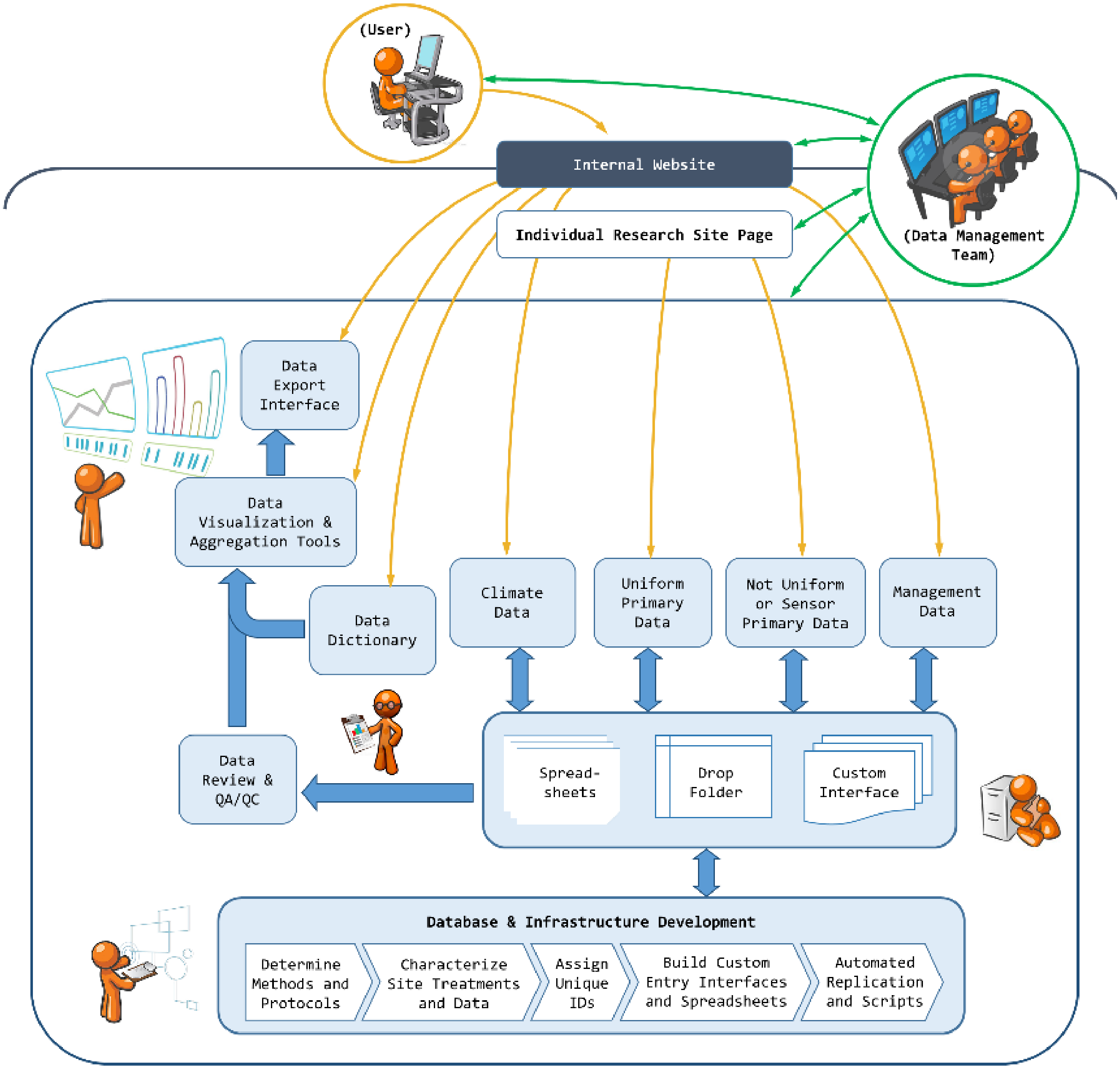

Supplementary Figure 1. Cloud-entry interfaces and overall workflow of team members and data personnel to aggregate, curate, and publish experimental data. Arrows show interactions and whether they are expected to be uni- or multi-directional.

Supplementary Table 1. Summary statistics (number of observations, mean, Q1, median, and Q3) for the variables not included in Table 3.

**Table**	**Variable (units)**	**n**	**mean**	**Q1**	**median**	**Q3**
Agronomic	leaf_area_index† (unitless)	422	4.3	3.7	4.3	4.9
Agronomic	grain_biomass† (kg/ha)	348	8395	4693	9621	11369
Agronomic	corn_cob_biomass (kg/ha)	146	1269	1126	1279	1418
Agronomic	forage_biomass† (kg/ha)	90	1399	851	1234	1711
Agronomic	vegetative_biomass† (kg/ha)	73	6224	4667	6052	7248
Agronomic	grain_total_C† (kg C/ha)	72	4160	3696	4183	4557
Agronomic	vegetative_total_C† (kg C/ha)	71	2748	2041	2652	3132
Agronomic	vegetative_total_N† (kg N/ha)	71	44.2	35.0	41.0	52.1
Agronomic	corn_cob_total_C (kg C/ha)	68	575	493	558	672
Agronomic	corn_cob_total_N (kg N/ha)	68	6.2	4.9	6.1	7.4
Agronomic	whole_plant_biomass† (kg/ha)	32	21393	18432	21340	23942
Agronomic	whole_plant_total_N† (kg N/ha)	32	245	208	236	280
Drainage	discharge (m3/day)	47275	241.04	0.00	0.48	46.55
Drainage	nitrate_N_removed (kg N)	8886	0.42	0.00	0.00	0.35

Water Quality	total_N_unfiltered_concentration (mg N/l)	4411	6.8	1.4	2.5	8.0
Water Quality	pH (unitless)	2858	7.8	7.5	7.8	8.1
Water Quality	total_P_filtered_concentration (ug P/l)	2510	107	0	58	130
Water Quality	water_ec (uS/cm)	2316	1606	730	1241	2300
Water Quality	total_N_filtered_concentration (mg N/l)	1726	5.1	1.0	2.0	6.2
Water Quality	ortho_P_unfiltered_concentration (ug P/l)	212	36	5	8	24
Soil physicochemical properties	NH4_concentration (mg NH4-N/kg)	1572	1.7	0.5	0.9	2.0
Soil physicochemical properties	percent_clay (%)	1084	35	25	34	44
Soil physicochemical properties	percent_sand (%)	1084	15	8	12	18
Soil physicochemical properties	percent_silt (%)	1084	51	40	50	63
Soil physicochemical properties	cec (cmol/kg)	1013	26.7	16.1	23.0	30.4
Soil physicochemical properties	soc (%)	958	1.7	0.9	1.4	2.4
Soil physicochemical properties	total_N (%)	935	0.1	0.1	0.1	0.2
Soil physicochemical properties	pH_water (unitless)	641	6.8	6.2	6.7	7.6
Soil physicochemical properties	pH_salt (unitless)	489	5.8	5.3	5.9	6.4
Soil physicochemical properties	som (g/kg)	465	22	17	21	24
Soil physicochemical properties	Ca_amount (kg Ca/ha)	464	4800	3879	4680	5597

Soil physicochemical properties	K_amount (kg K/ha)	464	307	214	298	377
Soil physicochemical properties	Mg_amount (kg Mg/ha)	464	723	379	538	992
Soil physicochemical properties	P_B1_amount (kg P/ha)	443	45	15	28	66
Soil physicochemical properties	K_concentration (g K/kg)	304	161	111	139	190
Soil physicochemical properties	Ca_concentation (g Ca/kg)	301	3134	2408	2960	3490
Soil physicochemical properties	Mg_concentration (g Mg/kg)	301	655	458	586	733
Soil physicochemical properties	NO3_amount (kg NO3-N/ha)	271	56	18	34	70
Soil physicochemical properties	neutralizable_acidity (cmol/kg)	249	3.4	1.5	3.0	5.5
Soil physicochemical properties	lime_index (unitless)	214	67.2	66.2	67.5	68.6
Soil physicochemical properties	salinity_paste (dS/m)	183	3.1	0.6	1.4	5.1
Soil physicochemical properties	sar (unitless)	183	1.9	0.9	1.5	2.6
Soil physicochemical properties	P_M3_concentration (ug P/kg)	144	23	2	11	38
Soil physicochemical properties	P_B1_concentration (ug P/kg)	131	23	5	15	36
Soil physicochemical properties	Ca_saturation (%)	104	65.7	56.9	66.6	74.5
Soil physicochemical properties	K_saturation (%)	104	1.3	1.0	1.2	1.6
Soil physicochemical properties	Mg_saturation (%)	104	18.7	15.1	18.6	21.3
Soil physicochemical properties	hydraulic_conductivity (cm/hr)	57	1.8	0.2	0.3	

Soil physicochemical properties	Na_concentration (g Na/kg)	50	192	126	181	242
Soil physicochemical properties	salinity_water (dS/m)	50	0.8	0.6	0.8	1.1
Soil physicochemical properties	Na_saturation (%)	30	3.6	3.2	3.9	4.4
Soil physicochemical properties	NH4_amount (kg NH4-N/ha)	25	17	11	16	19
Soil physicochemical properties	infiltration_rate (cm/hr)	24	1.4	1.3	1.4	1.5
Weather	air_temp_avg (deg C)	46299	10.5	1.7	11.8	20.4
Weather	wind_speed (m/s)	41458	4.7	1.8	3.3	5.9
Weather	relative_humidity (%)	40543	73.8	65.7	74.3	82.8
Weather	wind_direction (deg)	22190	197.7	128.5	205.0	279.9
Weather	et (mm)	13214	2.7	1.0	2.3	4.1
Weather	dew_point_temp_avg (deg C)	2555	2.9	-5.0	3.1	14.0

[†]Measurements are reported across crops including corn, soybean, wheat, popcorn, sugarbeet, cereal rye and sorghum-sudangrass.
